# Optimizing crop clustering to minimize pathogen invasion in agriculture

**DOI:** 10.1038/s41598-025-30635-9

**Published:** 2025-12-13

**Authors:** Yevhen F. Suprunenko, Christopher A. Gilligan

**Affiliations:** Department of Plant Sciences, University of Cambridge, Downing Street, Cambridge, CB2 3EA UK

**Keywords:** Computational biology and bioinformatics, Ecology, Ecology, Mathematics and computing, Plant sciences

## Abstract

The initial rate of pathogen invasion in crops is influenced by the spatial clustering of susceptible crops and the characteristics of pathogen dispersal. Previous studies have shown that various degrees of crop clustering can effectively reduce this invasion rate. However, the optimal degrees of clustering that minimize pathogen invasion have not previously been identified. This study aims to determine analytically the range of crop clustering that minimizes the initial rate of pathogen invasion. We studied artificial agricultural landscapes with crop areas arranged in identical square clusters on a regular square lattice. For pathogen dispersal, we used several common dispersal kernels, including Gaussian, negative exponential, and power-law. The optimal degree of clustering, defined by cluster size and separation distance, was calculated using a new analytical approximation for the pathogen invasion rate, which showed strong agreement with computer simulations. Additionally, we analysed a realistic cassava landscape at risk of invasion by cassava brown streak virus. We identified a range of optimal cluster sizes and corresponding separation distances that minimize pathogen invasion rates for various dispersal kernels and landscapes with clusters of crop fields arranged on a regular square lattice. The methods can be extended to other geometrical configurations, such as long narrow fields. Using a cassava landscape as an example, we show how optimal crop clustering strategies can be derived to mitigate the potential invasion of cassava brown streak virus. The methods provides analytical insights that can help farmers and agricultural planners to optimize the spatial structure of agricultural landscapes to minimize initial pathogen invasion rates.

**Supplementary Information:**

The online version contains supplementary material available at 10.1038/s41598-025-30635-9.

## Introduction

One of the key factors that determine how quickly a crop pathogen (or pest) invades and spreads across an agricultural landscape is the relationship between the pathogen’s dispersal and the spatial distribution of susceptible crops^1–13^. The rate of pathogen invasion can be slowed by adjusting the spatial configuration of host distribution relative to pathogen dispersal^1,5,8,9,11,12,14,15^. This can be achieved by reducing host aggregation^16–21^, growing resistant varieties^22^, or applying fungicides^7,23^.

Two simple measures of pathogen invasion are the infection rate, $\:r$, which denotes the early exponential growth rate of the number of infected fields at the onset of an epidemic, and the basic reproduction number $\:{R}_{0}$. The latter is defined as $\:{R}_{0}=r/\mu\:$, where $\:1/\mu\:$ represents the average infectious period of infected hosts.

Insights from theoretical^1,5,11,12^ and experimental^8,9^ studies indicate that pathogen invasion slows, and both $\:r$ and $\:{R}_{0}$ decline, as the host landscape becomes more dispersed (holding all other factors constant). While this might suggest that the minimal values of $\:r$ and $\:{R}_{0}$ would occur when fields of susceptible crops are evenly dispersed, recent theoretical work^24^ has shown that $\:r$ and $\:{R}_{0}$ can be minimized in agricultural landscapes with non-uniform spatial configurations. In these configurations, fields with susceptible crops can be aggregated into clusters (i.e. clustered) up to a threshold size.

**Fig. 1:**
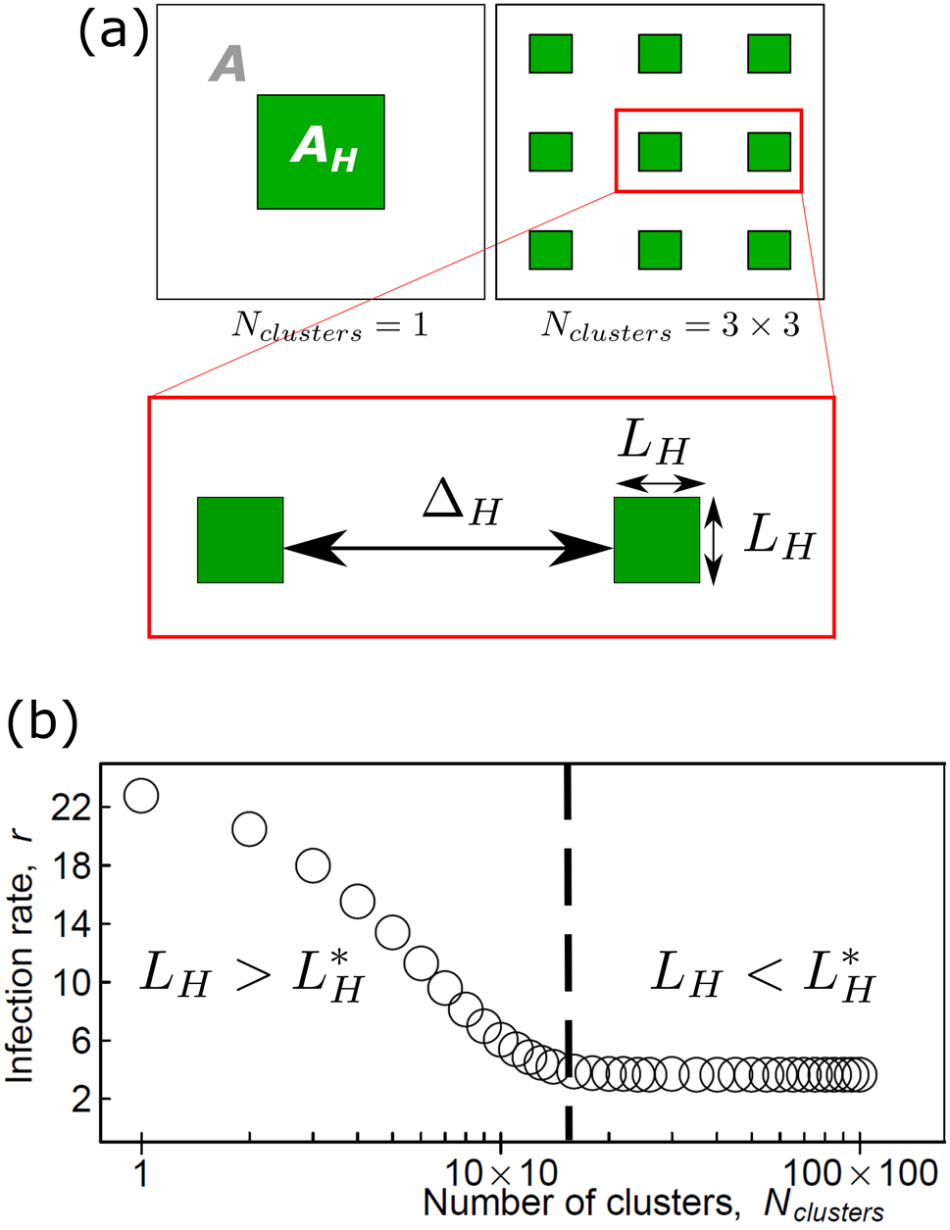
**a** An illustration of a crop area, $\:{A}_{H}$ (green), aggregated into one cluster ($\:{N}_{clusters}\:=\:1$) or nine clusters ($\:{N}_{clusters}\:=\:3\times\:3$), placed on a regular square grid within an agricultural landscape, $\:A$, where arbitrarily $\:{A}_{H}{A}^{-1}=0.15.$ The degree of clustering (aggregation) is characterised by the number of clusters, $\:{N}_{clusters}$, which determines the size ($\:{L}_{H}$) of a square cluster and the separation distance ($\:{{\Delta\:}}_{H}$) between clusters. **b** Open circles indicate the infection rate $\:r$ (the initial growth rate of the number of infected fields) estimated from simulations of an individual-based epidemic model initiated by a single randomly infected field within the landscapes of the type shown in Fig. 1a^24^; Supplementary Note 1). Reconfiguring the crop area $\:{A}_{H}$ into clusters of size $\:{L}_{H}$ smaller than the threshold size $\:{L}_{H}^{*}$ ($\:{L}_{H}<{L}_{H}^{*}$) reduces the infection rate from its maximum value (corresponding to $\:{N}_{clusters}\:=\:1$) to its minimum value (corresponding to $\:{N}_{clusters}\ge\:{N}_{clusters}^{*}$, where $\:{N}_{clusters}^{*}$ is the threshold number of clusters). Therefore, the infection rate of susceptible agricultural crop fields at the start of an epidemic can be minimized through various spatial reconfigurations of the landscape. The illustration is a modified version of Fig. 1 in Ref^24^. Computer codes for all figures in the current paper are available from Figshare^25^.

Suprunenko et al. (2025)^24^ illustrated this process using artificial landscapes in which a fixed area of susceptible crops was distributed in clusters on a regular square lattice (Fig. 1a). As the total crop area remained constant, the number of clusters ($\:{N}_{clusters}$) decreased as cluster size increased. The highest degree of clustering (i.e. the smallest $\:{N}_{clusters}$) associated with the minimum $\:r$ can be characterised by a threshold cluster size of width, $\:{L}_{H}^{*}$, and separation distance between clusters, $\:{{\Delta\:}}_{H}^{*}\:$(cf. Fig. 1b).

Understanding these threshold values, $\:{L}_{H}^{*}$ and $\:{{\Delta\:}}_{H}^{*}$, would be invaluable for agricultural planners in minimizing the risk of pathogen (or pest) invasion, given known dispersal characteristics.

Computer simulation of epidemic models on simulated landscapes can, in principle, be used to estimate the threshold size $\:{L}_{H}^{*}$ and separation distance $\:{{\Delta\:}}_{H}^{*}$ for a given pathogen dispersal kernel^15,20,21,26,27^. This involves analysing epidemics on host landscapes with many different spatial configurations to identify the degrees of host clustering that minimize $\:r$ (or $\:{R}_{0}$). However, constructing realistic epidemic spread models for each new pest or pathogen can be challenging, and considering numerous spatial configurations is computationally demanding and potentially inefficient.

Here, we explore an alternative approach to identify landscapes that minimise the initial rates of pathogen invasion using an analytical approximation for the initial infection rate, $\:r$. We test this analytical approximation against empirical infection rates derived from computer simulations of an individual-based model (IBM) for pathogen invasion and spread through a host landscape. Using the analytical approximation for $\:r$, we derive values for the threshold size $\:{L}_{H}^{*}$ and separation distance $\:{{\Delta\:}}_{H}^{*}$ for a range of generic host landscapes and commonly-used dispersal kernels, including Gaussian, negative exponential, and power-law kernels, among others. We illustrate a potential application of this approach, motivated by the spread of cassava brown streak virus (CBSV), which threatens cassava production in sub-Saharan Africa^28–30^. Using a dispersal kernel for CBSV estimated by Godding et al.^31^ and a section of a cassava landscape provided by Szyniszewska (2020)^32^, we present examples of maps showing estimates of local threshold sizes and separation distances for clusters of cassava fields that minimise infection rate.

## Methods

### Mathematical approximation for infection rate

We consider a susceptible-infected (SI) epidemiological compartmental individual-based model (IBM) for the invasion and spread of a pathogen through an agricultural landscape partially occupied by susceptible host crops. An analytical expression for the infection rate, $\:r$, for this IBM has been variously derived in several studies, including those by Bolker (1999)^1^, North and Godfray (2017)^12^, van den Bosch et al. (2024)^33^ and others^11,24,34,35^. For example, consider the approximation from Suprunenko et al. (2025)^24^:

1 $$\:r=\beta\:\times\:\left({n}_{S}+\frac{{n}_{S}}{{n}^{2}}\underset{0}{\overset{\frac{\sqrt{A}}{2}}{\int\:}}2\pi\:xb\left(x\right)g\left(x\right)\text{d}x\right),$$

where $\:b\left(x\right)$ is the pathogen dispersal kernel as a function of distance $\:x$, and $\:\beta\:$ is the infection rate per contact density (i.e. the spatial density of susceptible hosts that an initial infected field can reach through the dispersal kernel $\:b\left(x\right)$). The host landscape is assumed to be a square plot of land with area $\:A$ partially occupied by land containing a susceptible crop consisting of $\:N$ sufficiently small, identical individual fields with area $\:{A}_{0}$. Each field is treated as an individual ‘host’, described as a point-like object located at the centre of a field. The initial densities $\:{n}_{S}=(N-1){A}^{-1}$ and $\:n=N{A}^{-1}$ are the densities of susceptible hosts and all hosts respectively. The total area occupied by hosts is $\:{A}_{H}$, i.e. $\:{A}_{H}={A}_{0}N$, where $\:{A}_{H}\le\:A$. The function, $\:g\left(x\right)$, denotes the second order spatial cumulant (also known as a truncated correlation function or autocovariance, see e.g.^36,37^, that characterises the spatial structure of a host landscape.

The expression inside the brackets in Eq. (1) represents the spatial density of contacts with susceptible hosts (i.e. fields) from a randomly located initial infected field. Similarly, van den Bosch et al. (2024)^33^ described an equivalent product, $\:b\left(x\right)g\left(x\right)$ (cf. Eq. 1), as one that “describes how a pathogen ‘sees’ the host population around an infected host individual”^33^. Based on these insights, we construct an explicit expression for the spatial density of contacts to replace the expression in brackets in Eq. (1).

Our approach differs from conventional IBMs, which consider individual hosts as point-like entities and calculate the infection rate (1) based on their spatial distribution. In contrast, we explicitly consider individual hosts (i.e. fields) as square plots of cropped land with area $\:{A}_{0}$, and estimate the infection rate using the spatial distribution of these land polygons. We denote the spatial density of fields (measured as the number of fields per area) at location $\:\varvec{x}$ as $\:n\left(\varvec{x}\right)$, defining $\:n\left(\varvec{x}\right)={A}_{0}^{-1}$ when a point $\:\varvec{x}$ lies within a field, and $\:n\left(\varvec{x}\right)=0$ when$\:\:\varvec{x}$ is outside a field. A pathogen at location $\:\varvec{x}$ can infect directly the density of fields $\:{n}_{b}\left(\varvec{x}\right)$, where

$$\:{n}_{b}\left(\varvec{x}\right)=\int\:b\left(\left|{\varvec{x}}_{0}-\varvec{x}\right|\right)n\left({\varvec{x}}_{0}\right)\text{d}{\varvec{x}}_{0},$$

and the integration is over the entire landscape. Averaging $\:{n}_{b}\left(\varvec{x}\right)$ over all possible locations, $\:\varvec{x}$, within the landscape leads to the quantity $\:\stackrel{-}{n}$,

$$\:\stackrel{-}{n}\:=\left(\int\:{n}_{b}\left(\varvec{x}\right)n\left(\varvec{x}\right)d\varvec{x}\right)/\left(\int\:n\left(\varvec{y}\right)\text{d}\varvec{y}\right),$$

that can be interpreted as the spatially averaged density of contacts (with susceptible fields) that can result in infection from an initial randomly infected field. Therefore, the expression in brackets in Eq. (1) can be estimated by the density $\:\stackrel{-}{n}$, i.e.

2 $$\:r=\beta\:\times\:\stackrel{-}{n}.$$

Further to this, we make the following two simplifying approximations.

*Approximation 1*: Here, we develop an approximation for Eq. (2) when there is a high degree of aggregation of the target crop within a landscape (i.e. when $\:{N}_{clusters}$ is small): $\:r\approx\:\beta\:\times\:{\stackrel{-}{n}}_{1}\left({L}_{H}\right)$, where $\:{\stackrel{-}{n}}_{1}\left({L}_{H}\right)$ is derived as follows. We assume that the crop area is distributed into identical $\:{L}_{H}\times\:{L}_{H}$ square clusters on a regular square lattice, as illustrated in Fig. 1a. The total number of clusters is $\:{N}_{clusters}$. We denote the spatial density of crops within a single cluster as $\:{n}_{1}\left(\varvec{x}\right)$, where $\:{n}_{1}\left(\varvec{x}\right)={A}_{0}^{-1}$ if $\:0\le\:{x}_{1}\le\:{L}_{H}\:\text{a}\text{n}\text{d}\:0\le\:{x}_{2}\le\:{L}_{H}$ (where $\:{x}_{1}$ and $\:{x}_{2}$ are the coordinates in a two-dimensional Cartesian coordinate system), and $\:{n}_{1}\left(\varvec{x}\right)=0$ otherwise. Given a high degree of clustering, i.e. when all hosts are aggregated into a small number of large clusters, we estimate $\:r$ on a single cluster and use $\:{n}_{1}\left(\varvec{x}\right)$ instead of $\:n\left(\varvec{x}\right)$ in the expression (2), obtaining $\:{\stackrel{-}{n}}_{1}$ instead of $\:\stackrel{-}{n}$, where.

$\:{\stackrel{-}{n}}_{1}\:=\left(\int\:{n}_{b}\left(\varvec{x}\right){n}_{1}\left(\varvec{x}\right)d\varvec{x}\right)/\left(\int\:{n}_{1}\left(\varvec{y}\right)\text{d}\varvec{y}\right).$Using straightforward algebraic transformations, shown in the electronic supplementary material (Supplementary Note 2), the expression for $\:{\stackrel{-}{n}}_{1}$ is simplified further:

3 $$\:{\stackrel{-}{n}}_{1}\left({L}_{H}\right)=4{L}_{H}^{2}{A}_{0}^{-1}\underset{0}{\overset{1}{\int\:}}\underset{0}{\overset{1}{\int\:}}\text{d}x\text{d}y\:b\left({L}_{H}\sqrt{{x}^{2}+{y}^{2}}\right)(1-x)(1-y).$$

The quantity $\:{\stackrel{-}{n}}_{1}$ denotes the averaged density of fields within a single cluster that a randomly introduced infected field within that cluster can infect directly via a dispersal kernel $\:b\left(x\right)$. The upper bound of the quantity $\:{\stackrel{-}{n}}_{1}$ is $\:{A}_{0}^{-1}$, i.e. the density of crops within a single cluster.

*Approximation 2*: Here, we develop an approximation for Eq. (2) when there is a low degree of clustering in the host landscape: $\:r\approx\:\beta\:\times\:n$. We assume that the pathogen dispersal kernel, $\:b\left(x\right)$, is sufficiently long-ranged relative to an individual field of area $\:{A}_{0}$, i.e. the characteristic dispersal length, $\:a$, should be larger than the size of an individual field $\:(a>\sqrt{{A}_{0}})$. Consequently, the minimum value of the infection rate can be approximated as $\:{r}_{min}=\beta\:\times\:n$, where $\:n$ is the spatial density of all $\:N$ fields of a host crop within the landscape with area $\:A$, $\:n=N{A}^{-1}$. For a low degree of clustering, we therefore use $\:r\approx\:{r}_{min}$.

Finally, we combine expressions for the infection rate within a single cluster, $\:\beta\:\times\:{\stackrel{-}{n}}_{1}\left({L}_{H}\right)$ (approximation 1), and its minimal value, $\:\beta\:\times\:n$ (approximation 2) noting that the larger estimate should be used between approximations 1 and 2. Indeed, when the crop is highly aggregated within a landscape, the estimate of $\:r$ is given by $\:\beta\:{\stackrel{-}{n}}_{1}\left({L}_{H}\right)$ where $\:\beta\:{\stackrel{-}{n}}_{1}\left({L}_{H}\right)>\beta\:n$; while for a low degree of crop aggregation the estimate of $\:r$ is given by $\:\beta\:n$ when $\:\beta\:n>\beta\:{\stackrel{-}{n}}_{1}\left({L}_{H}\right)$. Therefore, using the maximum of two estimates, we use the following approximation for $\:r$, denoted as $\:{r}_{approximate}$:

4 $$\:{r}_{approximate}\:=\beta\:\times\:\text{m}\text{a}\text{x}\left[{\stackrel{-}{n}}_{1}\left({L}_{H}\right),n\right].$$

It is important to stress that this approximation is applicable to dispersal kernels, $\:b\left(x\right)$, that are decreasing functions of distance $\:x$. For dispersal kernels that do not decrease monotonically with distance, such as inverse Gaussian (Wald) or lognormal dispersal kernels (see^38^) the infection rate does not necessarily decrease as the number of clusters ($\:{N}_{clusters})$ increases.

### Computer simulations of an individual-based model

**Fig. 2:**
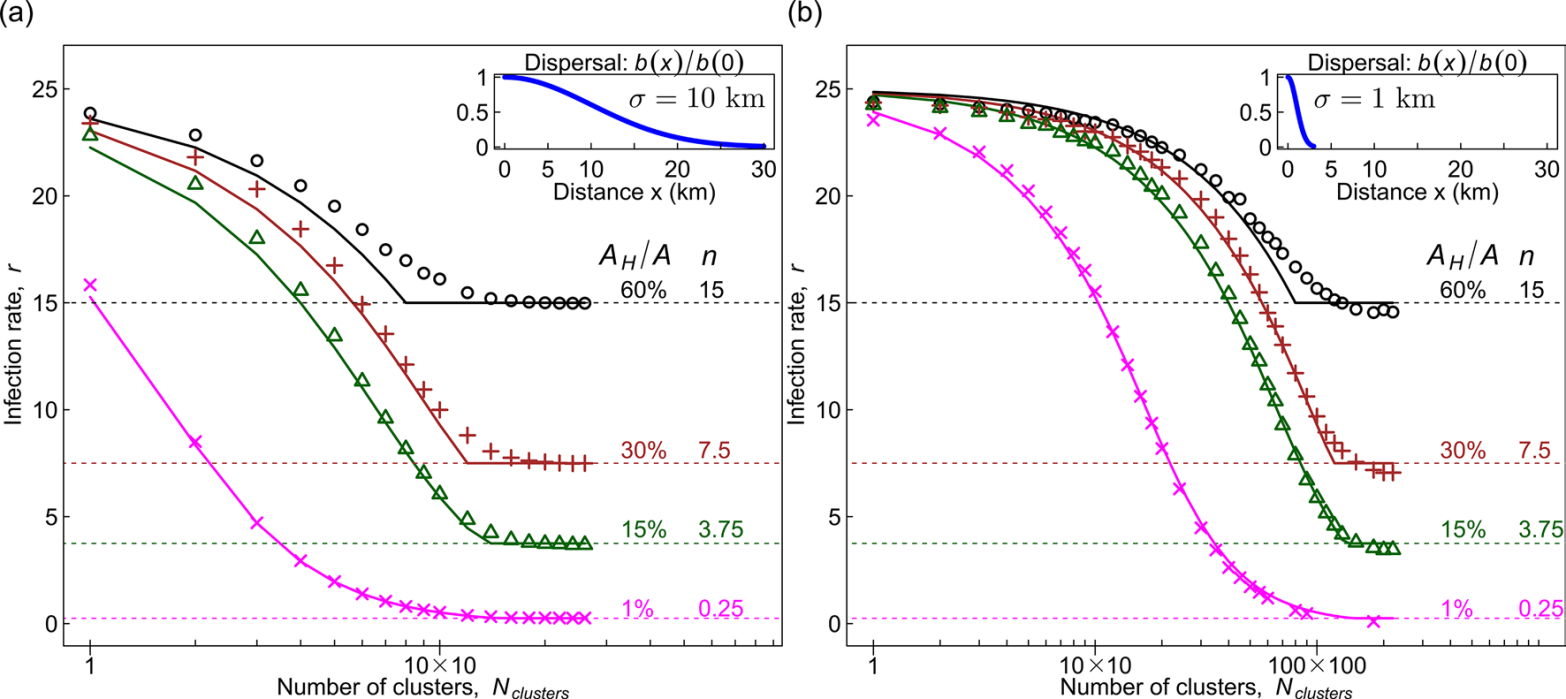
The behaviour of the analytically approximated infection rate, $\:{r}_{approximate}$, (lines corresponding to Eq. 4) as a function of the degree of clustering of susceptible crops is compared with estimates of infection rate $\:r$ (symbols) inferred from computer simulations of an individual-based model (see Methods). Results are shown for different landscapes and for two different dispersal kernels given by a Gaussian function with standard deviation: **a** $\:\sigma\:=10$ km, and **b** $\:\sigma\:=1$ km. The quantity $\:{A}_{H}{A}^{-1}$ denotes the fraction of the landscape occupied by susceptible fields; $\:n=N{A}^{-1}$ is the spatial density of all $\:N$ crop fields in the landscape with area $\:A$. Here, $\:\beta\:=1$, and the area of a unit field is $\:{A}_{0}=0.04$ km^2^; the upper bound of the rate $\:r$ is given by $\:\beta\:{A}_{0}^{-1}=25$. The transition of $\:r$ from its maximum to minimum is captured by $\:{r}_{approximate}$ reasonably well, as quantified by the following measures. In both panels, the maximum deviation $\:\text{max}|r\:-\:{r}_{approximate}|$ is 0.6, 0.9, 1.3 and 2.0 for $\:{A}_{H}/A=1,\:15,\:30$ and 60, respectively. These deviations correspond to 3.7%, 4.5%, 8.2% and 22% of the total range $\:\text{max}\left(r\right)-\text{min}\left(r\right)$ for the same values of $\:{A}_{H}/A$.

To test the approximation derived above, we compare the performance of Eq. (4) with empirical rates for *r* at the start of an epidemic, originally derived from computer simulations of an individual-based model of pathogen invasion in an agricultural landscape^24^. For convenience, details of the individual-based model are summarised in Supplementary Note 1. Exploratory testing indicated that the transition of $\:r$ from its maximum to its minimum value captured by $\:{r}_{approximate}$ (Eq. 4) sufficiently well (see caption of Fig. 2); therefore, we proceed to use this approximation.

In Fig. 2 we used the data^39^ from computer simulations of individual-based model of pathogen invasion and spread in agricultural landscapes that were taken from^24^. Here we test a different analytical approximation from^24^ for identifying optimal clustering to reduce initial invasion rate, inspired by the original more computationally intensive method in^24^ that uses spatial cumulants to assess the impact of general landscape structures.

### Mathematical approximation for threshold size and separation distance

We define the threshold size $\:{L}_{H}^{\text{*}}$ as the value of the argument of the function $\:{\stackrel{-}{n}}_{1}\left({L}_{H}\right)$ in Eq. (4) such that.

5 $$\:{\stackrel{-}{n}}_{1}\left({L}_{H}^{\text{*}}\right)=n.$$

Using the definition $\:n=N{A}^{-1}={A}_{H}{\left(A{A}_{0}\right)}^{-1}$, the condition (5) can be re-written as

6 $$\:{\stackrel{-}{n}}_{1}\left({L}_{H}^{\text{*}}\right){A}_{0}={A}_{H}{A}^{-1}.$$

The right-hand side of Eq. (6) is a dimensionless parameter $\:{A}_{H}{A}^{-1}$ denoting the fraction of the landscape occupied by susceptible fields. For the most typical dispersal kernels, $\:b\left(x\right)$, characterized by a scale parameter $\:a$ (see Supplementary Note 3, Table S1), the left-hand side of Eq. (6) depends on the dimensionless parameter $\:{L}_{H}^{\text{*}}{a}^{-1}$ explicitly (see electronic supplementary material, Supplementary Note 4 for details). Therefore, for a landscape characterized by $\:{A}_{H}{A}^{-1}$, the Eq. (6) determines $\:{L}_{H}^{\text{*}}$ in units of the scale parameter $\:a$ that characterises the pathogen dispersal kernel $\:b\left(x\right)$. The threshold separation distance $\:{{\Delta\:}}_{H}^{\text{*}}$ is then determined (on a regular square lattice, Fig. 1a) by $\:{L}_{H}^{\text{*}}$ and $\:{A}_{H}{A}^{-1}$:.

7 $$\:{{\Delta\:}}_{H}^{\text{*}}={L}_{H}^{*}\times\:\left(\sqrt{{A/A}_{H}}-1\right).$$

Parameters and functions used in the current paper are summarized in Table 1.

**Table 1:** Parameters used in the model, together with characteristic lengths of pathogen dispersal and host landscape and their threshold values.

Symbol	Description
Model parameters	
$\:A$	Entire area of the landscape, a square plot of land.
$\:{A}_{0}$	Area occupied by a single field of host crop (a square plot of cropped land).
$\:N$	Number of host crop fields within the landscape.
$\:{A}_{H}$	Total area occupied by fields (host crop), $\:{A}_{H}={A}_{0}\times\:N;\:\:{A}_{H}\le\:A.$
$\:n$	Density of all host crop fields within a landscape,$\:n=N/A$.
$\:{n}_{S}$	Density of susceptible fields within a landscape, assuming a single initial infected field, $\:{n}_{S}=(N-1)/A$.
$\:b\left(x\right)$	Pathogen dispersal kernel.
$\:\sigma\:$	Standard deviation of a Gaussian function.
$\:\beta\:$	Infection rate per contact density.
$\:r$	Rate of infection of susceptible fields at the start of an epidemic, Eq. (1).
Spatial scales and degree of aggregation of a host landscape	
$\:a,{b}_{0}$	Scale parameter ($\:a$) and a shape parameter ($\:{b}_{0}$) of a pathogen dispersal kernel, see Supplementary Material, Supplementary Note 3, Table S1.
$\:{L}_{H},{{\Delta\:}}_{H}$	Cluster size ($\:{L}_{H}$) and separation distance ($\:{{\Delta\:}}_{H}$) characterising a host crop landscape, Fig. 1a.
$\:{N}_{clusters}$	Number of clusters of cropped fields within the landscape, $\:{N}_{clusters}={A}_{H}/{L}_{H}^{2}=A/{\left({L}_{H}+{{\Delta\:}}_{H}\right)}^{2}$. It can be seen as a measure of the degree of aggregation of a host landscape.
Threshold values	
$\:{L}_{H}^{*},{{\Delta\:}}_{H}^{*}$ $\:{N}_{clusters}^{*}$	Threshold values of cluster size ($\:{L}_{H}^{*}$) and separation distance ($\:{{\Delta\:}}_{H}^{*}$), Fig. 1c, expressed in units of the scale parameter$\:a$ and calculated from Eqs. (6) and (7).$\:{N}_{clusters}^{*}$is the corresponding threshold value of the number of clusters.

### Cassava landscape and CBSV

We illustrate the analytical approach with a real-life example: the invasion of CBSV in a cassava landscape. Godding et al. (2023)^31^ developed and parameterised an epidemic spread model for CBSV with a pathogen dispersal kernel defined on a raster with a 1 km spatial resolution, imposed by the spatial resolution of available host distribution data^32^. The CBSV dispersal, determined in^31^ by $\:\beta\:$ and a function $\:K\left({d}_{ij}\right)$ of distance $\:{d}_{ij}$ between centroids of raster cells, accounts for the combined effect of short-distance CBSV spread via insect vectors and long-distance spread via movement of virus-infected planting materials^31^. Specifically, $\:K\left({d}_{ij}=0\right)=p$ and $\:K\left({d}_{ij}>0\right)=C{{d}_{ij}}^{-\alpha\:}$, where $\:p$ is the proportion of inoculum remaining in the source cell, $\:C$ is a normalization constant determined by $\:\sum\:_{j}K\left({d}_{ij}\right)=1$, and $\:\alpha\:$ is the kernel exponent. By fitting this model to surveillance data, Godding and co-authors^31^ derived posterior distributions for the parameters $\:\beta\:,$ $\:p$ and $\:\alpha\:$. Here, we use a radially symmetric, staircase-like version of the raster-based dispersal kernel from^31^. Using their results, we selected a power-law dispersal kernel $\:b\left(x\right)$, characterised by two parameters: the exponent $\:\alpha\:=3.75$ and $\:p=0.12$ taken from a region of relatively high probability density in posterior distributions estimated by Godding et al. (2023)^31^.

In common with^31^, we use CassavaMap^32^ as a source of mapped landscape data for cassava. The landscape data for cassava are derived^31^ from production statistics^32^ and resolved to 1 km^2^ raster cells across sub-Saharan countries that cultivate cassava. The analytical estimates (6)–(7) depend on the ratio of host crop area $\:{A}_{H}$ to the total area $\:A$. We express the landscape in terms of hectares of cassava fields per raster cell, noting that, according to^32^, no more than half of the area of each raster cell (approximately 50 hectares) can be allocated to cassava production. Therefore, the maximum value of $\:{A}_{H}{A}^{-1}$ in the cassava landscape data cannot exceed 0.5, i.e. here $\:{A}_{H}{A}^{-1}\le\:0.5$.

Finally, using the approach described above, we calculated $\:{L}_{H}^{*}$ and $\:{{\Delta\:}}_{H}^{*}$ (Fig. 4a-b, black curves) for a cassava landscape at risk of CBSV invasion.

## Results

Using the approximation for the rate $\:r$ (Eq. 4, Fig. 2), we identified a range of values of cluster size $\:{L}_{H}$ for which the infection rate, $\:r$, is minimized. Within this range, the largest cluster size, $\:{L}_{H}$, is referred to as a threshold cluster size $\:{L}_{H}^{*}$. Equation (6) determines the ratio $\:{L}_{H}^{*}/a$ of the threshold cluster size $\:{L}_{H}^{*}$ to the pathogen dispersal scale parameter $\:a$ for a given pathogen dispersal kernel $\:b\left(x\right)$ and the fraction $\:{A}_{H}{A}^{-1}$ of the landscape occupied by susceptible crops. Correspondingly, the threshold separation distance is calculated using Eq. (7).

### Artificial landscapes and commonly-used dispersal kernels

**Fig. 3:**
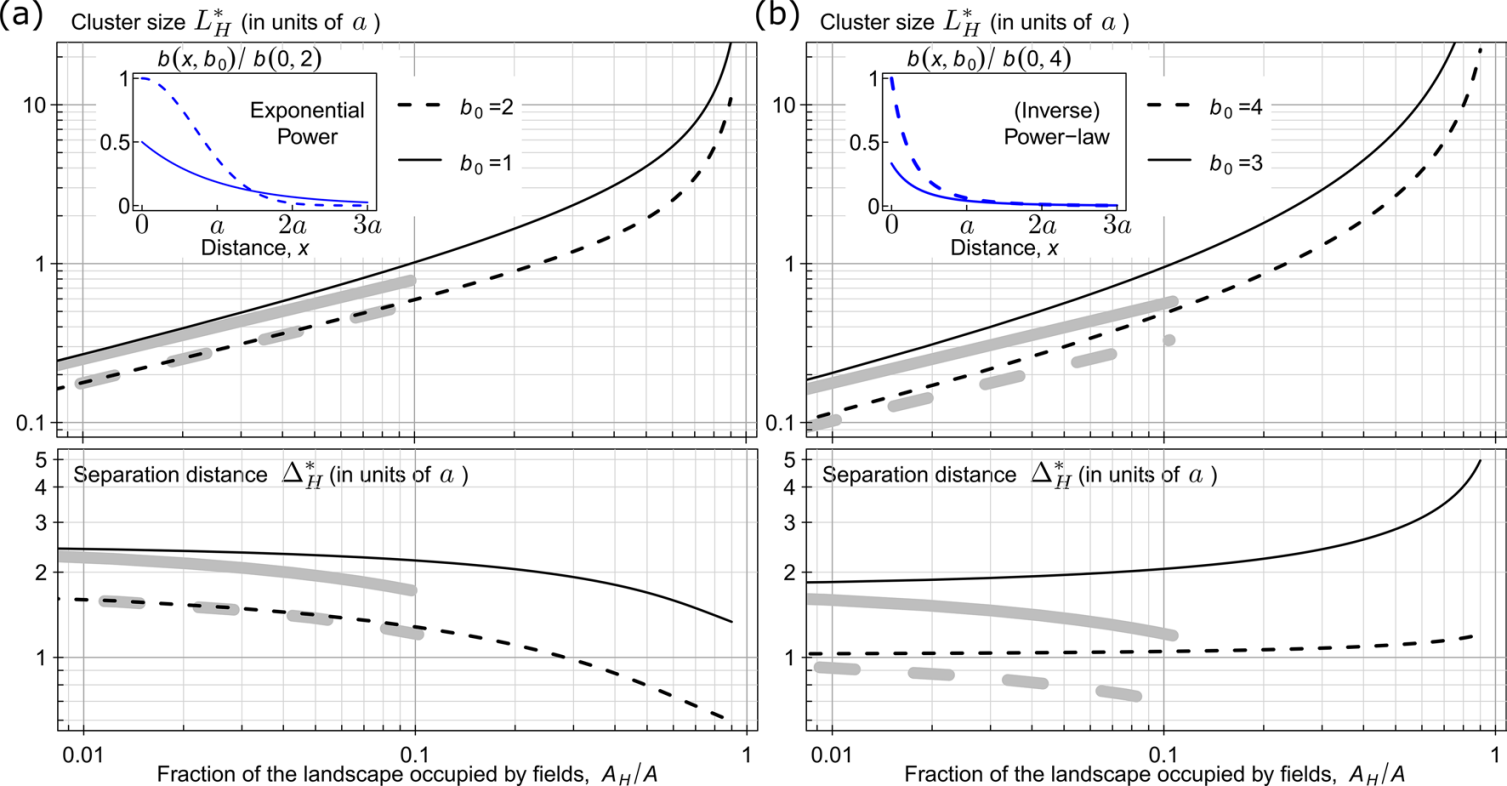
Threshold cluster size $\:{L}_{H}^{\text{*}}$ and threshold separation distance $\:{{\Delta\:}}_{H}^{\text{*}}$ identified for two types of dispersal kernel. **a** Results are shown for exponential power kernels, that correspond to the Gaussian kernel when $\:{b}_{0}=2$ and the negative exponential kernel when $\:{b}_{0}=1$, as shown in the inset of the panel. **b** Results are shown for inverse power-law kernels with exponents $\:{b}_{0}=3$ and $\:{b}_{0}=4$, as illustrated in the inset of the panel. For the exact analytical expressions for the dispersal kernels used here see Table S1 in Supplementary Note 3. In both panels, black curves represent numerical estimates from Eqs. (6) and (7), while grey curves at $\:{A}_{H}{A}^{-1}\le\:0.1$ represent explicit analytical estimates from Eqs. (7) and (8). The R and Mathematica codes used to calculate these results are available from Figshare^25^ and can be easily applied to other dispersal kernels.

Applying this approach to different dispersal kernels and landscapes, we first considered the Gaussian, negative exponential and power-law kernels – commonly used in epidemic spread studies^13,40,41^ – alongside several additional kernels detailed in Supplementary Note 3, Table S1. The results for the exponential and power-law kernels are summarised in Fig. 3, while the results for the other kernels from Table S1 are presented in the electronic supplementary material, Supplementary Note 4.

In addition to the numerical solution for $\:{L}_{H}^{*}$ from Eq. (6), we derived an explicit analytical estimate for $\:{L}_{H}^{*}$ when the value of $\:{A}_{H}{A}^{-1}$ is small. In this case, the value of $\:{L}_{H}^{*}$ is also small. Therefore, using the approximation $\:b\left({L}_{H}^{*}\sqrt{{x}^{2}+{y}^{2}}\right)\approx\:b\left(0\right)$ in Eq. (6) we derived:

8 $$\:{L}_{H}^{*}\approx\:\sqrt{\frac{{A}_{H}}{b\left(0\right)A}},\:\:\text{w}\text{h}\text{e}\text{n}\:\:\:\frac{{A}_{H}}{A}\ll\:1.$$

The explicit analytical estimate (Eq. 8) depends only on the dispersal kernel at zero distance, $\:b\left(0\right)$, and ignores its distance-dependent component, $\:b(x\:>\:0)$. Consequently, the explicit analytical estimate of $\:{L}_{H}^{*}$ aligns better with the numerical solution for $\:{L}_{H}^{*}$ from Eqs. (6–7) when short-range dispersal dominates, as with Gaussian kernels (Fig. 3a), but less so for long-tailed kernels like the power-law (Fig. 3b). This is reflected in the closer agreement between the thin black (for the numerical solution, Eqs. 6–7) and the thick gray (explicit analytical estimate, Eq. 8) lines in Fig. 3a than in Fig. 3b. As the ratio $\:{A}_{H}/A$ increases, clusters move closer together, amplifying long-distance dispersal effects and reducing the accuracy of explicit analytical estimate (8), as seen in both panels.

We present an example of how results shown in Fig. 3 could inform stakeholders, including farmers and other agricultural planners, about the spatial structure of a host landscape. To keep the disease from spreading quickly at the start of an epidemic, it is important than no cluster of host crop fields is too large. Specifically, these clusters should not exceed a certain critical size (equal to $\:{L}_{H}^{\text{*}}\times\:{L}_{H}^{\text{*}}$). In addition, each cluster should be surrounded by a buffer zone – a strip of land of specific width (equal to $\:{{\Delta\:}}_{H}^{*}/2$) without susceptible host crops. Smaller clusters help limit disease spread within them, while buffer zones reduce the chance of transmission between clusters.

The same principle applies to clusters smaller than the critical size: any $\:{L}_{H}\times\:{L}_{H}$ clusters (where $\:{L}_{H}<{L}_{H}^{\text{*}}$) should be surrounded by a host-free buffer zone of width $\:{{\Delta\:}}_{H}/2$, where $\:{{\Delta\:}}_{H}={L}_{H}\left(\sqrt{{A/A}_{H}}-1\right)$ (cf. Eq. (7)). The buffer zones around different clusters should not overlap. If there are two clusters within the landscape, first cluster $\:{L}_{H}^{\left(1\right)}\times\:{L}_{H}^{\left(1\right)}$ and second cluster $\:{L}_{H}^{\left(2\right)}\times\:{L}_{H}^{\left(2\right)}$, where both sizes are below the threshold size, i.e. $\:{L}_{H}^{\left(1\right),\left(2\right)}\le\:{L}_{H}^{*}$, then the minimal suggested separation distance between these clusters equals the sum of the two corresponding buffer zones, i.e. $\:({{\Delta\:}}_{H}^{\left(1\right)}+{{\Delta\:}}_{H}^{\left(2\right)})/2$.

Although we considered the spatial distribution of the cropped area into square clusters on a regular square lattice, other geometrical configurations could be considered. For example, if the cropped area is distributed among long narrow fields (e.g. see^8^), we present the derivation of an explicit analytical estimate for $\:{L}_{H}^{*}$ in Supplementary Note 5, analogous to Eq. (8).

### Cassava landscape and an example of CBSV dispersal kernel

**Fig. 4:**
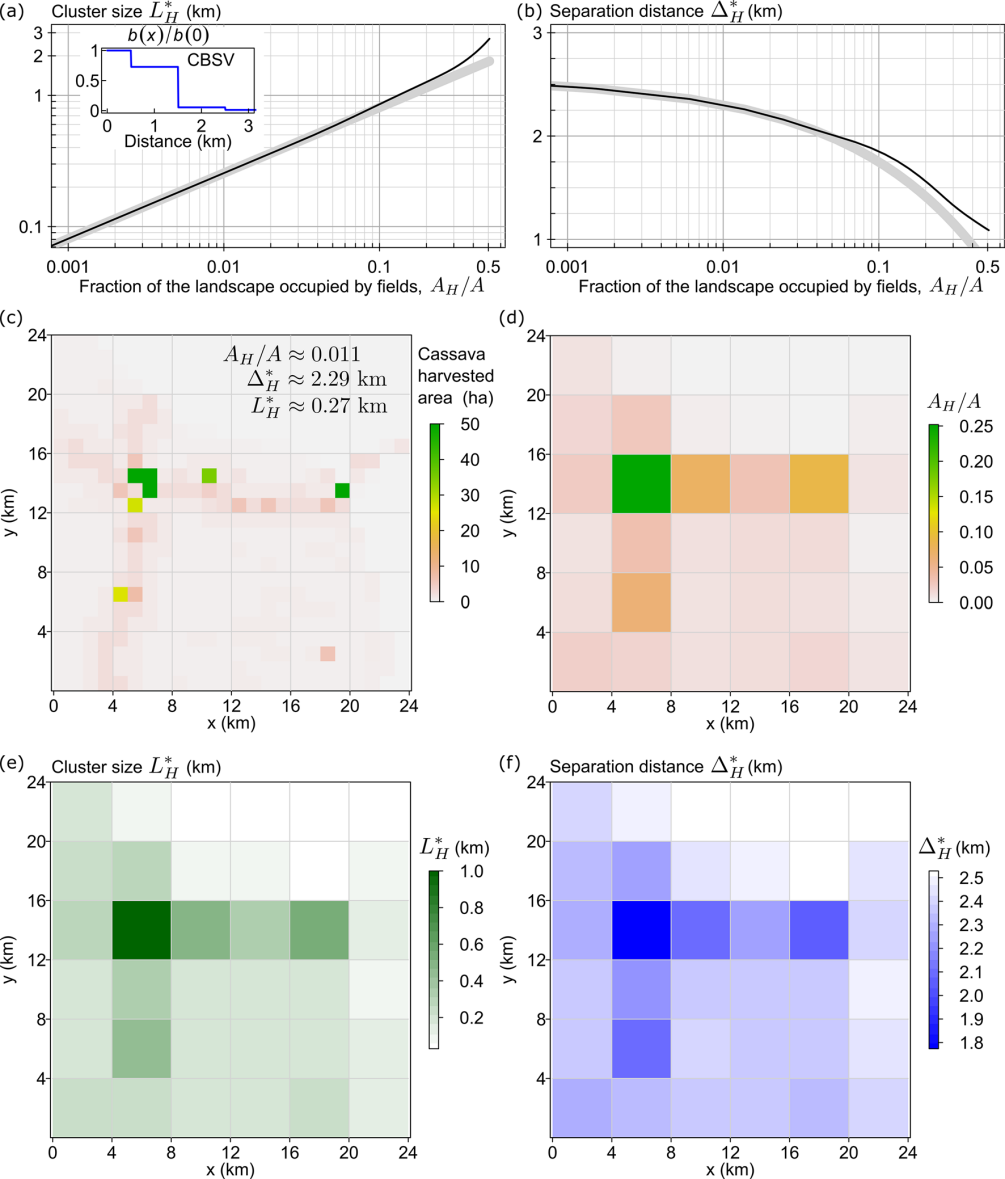
Threshold cluster size $\:{L}_{H}^{\text{*}}$ and threshold separation distance $\:{{\Delta\:}}_{H}^{\text{*}}$ identified for an example of CBSV dispersal kernel and a cassava landscape. **a**, **b** Threshold cluster size $\:{L}_{H}^{\text{*}}$ and separation distance $\:{{\Delta\:}}_{H}^{\text{*}}$ that minimise the initial infection rate $\:r$ as the fraction $\:{A}_{H}/A$ of the landscape occupied by the host crop increases. Black curves represent numerical estimates obtained from Eqs. (6) and (7), thick grey curves represent explicit analytical estimates from Eqs. (7) and (8). **c** The cassava landscape, given at 1 km spatial resolution. **d** The same cassava landscape at 4 km spatial resolution, shown in terms of the fraction $\:{A}_{H}/A$ of area occupied by the host crop. **e**, **f** Maps of $\:{L}_{H}^{\text{*}}$ and $\:{{\Delta\:}}_{H}^{\text{*}}$ calculated using information from panels (a), (b) and (d), showing how the crop area in each cell can be maximally clustered for the infection rate to remain minimal ($\:{r}_{min}=\beta\:n$, with the value of $\:n$ determined separately for each cell).

To demonstrate the application of the analytical approach to CBSV invasion in a cassava landscape, we used an area $\:A=24\times\:24$ km^2^ in an arbitrarily selected region on the border between Cameroon and the Central African Republic (Fig. 4c) derived from^32^. We calculated that cassava fields occupy 1.1% of this area (i.e. $\:{A}_{H}{A}^{-1}$ = 0.011). According to Fig. 4a and b, the corresponding threshold cluster size and separation distances are $\:{L}_{H}^{\text{*}}=0.27$ km and $\:{{\Delta\:}}_{H}^{\text{*}}=2.29$ km. Therefore, if it were logistically possible, our analysis suggests that organizing the crop area from Fig. 4c into clusters of $\:0.27$ km by $\:0.27$ km ($\:{L}_{H}^{\text{*}}\times\:{L}_{H}^{\text{*}}$), separated by $\:2.29$ km ($\:{{\Delta\:}}_{H}^{\text{*}}$), would result in the minimal infection rate, $\:{r}_{min}=\beta\:n$.

However, major redistributions of cropping patterns are unlikely to be practically feasible. Therefore, we considered a more practical approach by focusing on smaller 4 km by 4 km local areas where spatial reconfigurations of cropping patterns might potentially be possible. However, we emphasize that this is a hypothetical case to support the concept, as, in practice, there are many other major factors to be considered. We aggregated the original landscape to a 4 km spatial resolution (Fig. 4d). Using the fraction of area occupied by the host crop in each 4 km by 4 km cell, we assigned each cell a corresponding value of the threshold cluster size $\:{L}_{H}^{\text{*}}$ (Fig. 4e) and separation distance $\:{{\Delta\:}}_{H}^{\text{*}}$ (Fig. 4f).

For illustration, consider the cell in Fig. 4d–f located between 4 km and 8 km on the x axis, and between 12 km and 16 km on the y axis. Our analysis indicates that the infection rate would remain minimal if cassava fields were aggregated into a cluster approximately 1 km by 1 km in size, surrounded by a cassava-free buffer zone with a width of $\:{{\Delta\:}}_{H}^{*}/2\approx\:900$ m. In another cell, located between 16 km and 20 km on the x axis and between 12 km and 16 m on the y axis, the infection rate would remain minimal if cassava fields were aggregated into a cluster of 500 m by 500 m, surrounded by a cassava-free buffer zone with a width of $\:{{\Delta\:}}_{H}^{*}/2\approx\:1$ km.

Maps from Fig. 4e, f could in principle be used to inform decisions on where to plant new cassava fields without increasing the risk of early CBSV epidemic spread. If a cluster of size $\:{L}_{H}^{*}\times\:{L}_{H}^{*}$ already exists within a given 4 km by 4 km cell, new fields should not be placed within the recommended cassava-free buffer zone (with a width of $\:{{\Delta\:}}_{H}^{*}/2$) around the cluster to avoid increasing the infection rate. New fields can be planted near smaller clusters, but each smaller cluster of size $\:{L}_{H}\le\:{L}_{H}^{*}$ should be surrounded by a cassava-free buffer zone with a width of $\:{{\Delta\:}}_{H}/2$, where $\:{{\Delta\:}}_{H}={L}_{H}\left(\sqrt{{A/A}_{H}}-1\right)$.

## Discussion

To identify the range of optimal clustering of host crops that minimizes the pathogen invasion rate, the analytical approach presented in this paper integrates the intrinsic spatial characteristics of pathogen dispersal with the distribution of host crops. Considering a specific geometrical configuration of host landscapes, where susceptible crops are distributed in square clusters on a regular square lattice, the resulting threshold characteristics, cluster size $\:{L}_{H}^{*}$ and separation distance $\:{{\Delta\:}}_{H}^{\text{*}}$, are derived in units of the scale parameter$\:,\:a$, of a given pathogen dispersal kernel and depend on the fraction $\:{A}_{H}/A$ of the landscape occupied by susceptible crops. Consequently, this analytical approach is effective for a wide range of dispersal kernels and host landscapes.

We used two main approximations in deriving the analytical results. The first approximation was applied to landscapes where hosts were aggregated into a *small number* of clusters (i.e. where $\:r$ is a decreasing function of the number of clusters; see e.g. Fig. 2). In those cases, we estimated $\:r$ from a single cluster and the corresponding density $\:{\stackrel{-}{n}}_{1}$ (that is the spatially averaged density of contacts with susceptible crops within a single cluster that can result in infection from an initial randomly infected host), $\:r\approx\:\beta\:{\stackrel{-}{n}}_{1}$, (approximation 1, Eq. (3)).

In other landscapes, where crops were aggregated into a *large number* of clusters (i.e. where $\:r$ depends weakly on the number of clusters, see e.g. Figure 2), we used a second approximation and estimated $\:r$ assuming a constant density, $\:n$ (that is the density of crops within the entire landscape and therefore independent of the number of clusters), $\:r\approx\:\beta\:n$, (approximation 2).

Approximation 1 ($\:r\approx\:\beta\:{\stackrel{-}{n}}_{1}$) applies when clusters are sufficiently large and well-isolated, such that inter-cluster contacts are minimal. In this case, approximation 1 effectively considers each single cluster in isolation, neglecting inter-cluster contacts and therefore underestimating the actual value of $\:r$. In contrast, approximation 2 ($\:r\approx\:\beta\:n$) applies when numerous sufficiently small clusters are located close to each other. In this case, contacts may occur across many clusters, except the most distant ones. However, because approximation 2 assumes contacts with all hosts across the entire landscape, it overestimates the actual value of $\:r$. The intersection of the two approximations determines the threshold cluster size $\:{L}_{H}^{*}$, i.e. $\:\beta\:{\stackrel{-}{n}}_{1}\left({L}_{H}^{*}\right)=\beta\:n$ (Eq. 5), which is solved numerically to provide an exact value for $\:{L}_{H}^{*}$. The underestimation (due to approximation 1) of the left-hand side of Eq. 5 and the overestimation on the right-hand side (due to approximation 2) lead to an underestimation at the intersection point for the thresholds number of clusters, $\:{N}_{clusters}^{*}$ (Fig. 1c), and consequently, to an overestimation of $\:{L}_{H}^{*}$ due to the relationship $\:{L}_{H}^{*}=\sqrt{{A}_{H}/{N}_{clusters}^{*}}$. The resulting overestimation of $\:{L}_{H}^{*}$ provides a practical upper bound to the size of a cluster of crop fields that minimizes the risk of rapid spread during the initial stages of epidemics.

By using methods based on $\:r$ (or $\:{R}_{0}$ given the link in SIR models as $\:{R}_{0}=r/\mu\:$^24)^, our analysis focuses on short-term epidemiological dynamics. The analysis is therefore useful for predicting outcomes during the initial invasion but does not necessarily provide an accurate assessment of the long-term dynamics of an epidemic. However, in some cases, medium-term dynamics can still be characterised by $\:r$. For example, in our earlier work^24^ we demonstrated that “the analytical estimates of the infection rate correctly identified the relative ranking of the number of infected fields”^24^ for different landscapes in sub-Saharan Africa, using a six-month spread period as a proxy for medium-term dynamics. In general, the nonlinear dynamics, typical of many epidemics, mean that initial fast or slow rates of epidemic progress are often not sustained throughout the epidemic. For example, Brown and Bolker^5^ and Benincà et al.^42^ have shown how initially high values of $\:r$ or $\:{R}_{0}$ can result in a slow spread of pathogens at later stages of an epidemic, where there are trade-offs between local and long-range dispersal. Therefore, to generate robust predictions for long-term epidemiological dynamics in realistic agricultural landscapes, it is necessary to use computer simulations of epidemic spread models. For instance, Godding et al.^43^ modelled the 25-year spread of CBSV across sub-Saharan Africa, providing insights that go beyond what short-term analytical methods can offer.

The results of the current paper improve our understanding of the relationship between spatial scales of host distribution and pathogen dispersal, and their role in epidemic dynamics^7,44^. Specifically, the analytical results in Fig. 3 provide some general insights into the roles of the spatial scale parameter $\:a$ and the shape parameter $\:{b}_{0}$ (cf. Table S1 in Supplementary Note 3) of a dispersal kernel in epidemics. These insights can be used to quickly compare how different dispersal kernels affect the invasion speed of pathogens in the same host landscape. This opens the door to future work on identifying optimal host distributions under invasion by multiple pathogens with different dispersal kernels. In addition, dispersal kernels are often poorly known, which makes the analytical approach presented here particularly valuable. It enables rapid exploration of a broad family of potential kernels – even before detailed empirical estimates are available.

For example, from Eq. (6) and its dependence on the dimensionless parameter $\:{L}_{H}^{*}/a$, it follows that increasing the spatial scale parameter $\:a$ (i.e., increasing dispersal length) reduces the infection rate at the start of an epidemic towards its minimal value $\:\beta\:n$ (see Supplementary Note 6 for the details). Considering the role of the parameter $\:{b}_{0}$, Fig. 3a shows that the values of $\:{L}_{H}^{*}$ for the negative exponential kernel ($\:{b}_{0}=1$) are larger than for Gaussian kernel ($\:{b}_{0}=2$). This result indicates that, in the same host landscape, the infection rate $\:r$ at the start of an epidemic will be higher for the Gaussian kernel than for the negative exponential kernel, assuming $\:\beta\:$ is constant.

For ease of presentation, we have focused on pathogens, but the method can equally be applied to pest invasions where dispersal kernels are known or can be estimated. Ultimately, the analytical approach of this work has the potential to inform agronomic planning decisions^45,46^ to optimise the size and spacing between clusters of fields, thereby minimizing the initial infection rate of an invading pathogen. The results of this paper provide valuable insights into selecting methods to decelerate the initial spread of an invading pathogen across a landscape of susceptible crops. Future work could further develop this analytical approach to encompass the role of characteristic spatial scales associated with control measures^7,23^ on initial epidemic dynamics.

## Supplementary Information

Below is the link to the electronic supplementary material.

Supplementary Material 1

## Data Availability

The datasets analysed during the current study were sourced from the previously published work by Suprunenko et al. (2024) [39] and are available in the Figshare repository, https://doi.org/10.6084/m9.figshare.25804702.
